# Polymorphic Forms of Human Cytomegalovirus Glycoprotein O Protect against Neutralization of Fibroblast Entry by Antibodies Targeting Epitopes Defined by Glycoproteins H and L

**DOI:** 10.3390/v14071508

**Published:** 2022-07-09

**Authors:** Li He, Scott Taylor, Catherine Costa, Irene Görzer, Julia Kalser, Tong-Ming Fu, Daniel Freed, Dai Wang, Xiaohong Cui, Laura Hertel, Michael A. McVoy

**Affiliations:** 1 Department of Microbiology & Immunology, Virginia Commonwealth University, Richmond, VA 23298, USA; hel3@vcu.edu; 2 School of Medicine, Virginia Commonwealth University, Richmond, VA 23298, USA; taylorms3@mymail.vcu.edu (S.T.); costac@mymail.vcu.edu (C.C.); 3 Center for Virology, Medical University of Vienna, 1090 Vienna, Austria; irene.goerzer@meduniwien.ac.at (I.G.); julia.kalser@roteskreuz.at (J.K.); 4 Texas Therapeutic Institute, The University of Texas Health Science Center at Houston, Houston, TX 77030, USA; tong-ming.fu@uth.tmc.edu; 5 Merck & Co., Inc., Rahway, NJ 07065, USA; dan_freed@merck.com (D.F.); dai_wang@merck.com (D.W.); 6 Department of Anatomy, Virginia Commonwealth University, Richmond, VA 23298, USA; xiaohong.cui@vcuhealth.org; 7 Department of Pediatrics, University of California San Francisco, Oakland, CA 94609, USA; laura.hertel@ucsf.edu; 8 Department of Pediatrics, Virginia Commonwealth University, Richmond, VA 23298, USA

**Keywords:** cytomegalovirus, neutralizing antibody, hyperimmune globulin, glycoprotein O, polymorphism, immune evasion

## Abstract

Human cytomegalovirus (CMV) utilizes different glycoproteins to enter into fibroblast and epithelial cells. A trimer of glycoproteins H, L, and O (gH/gL/gO) is required for entry into all cells, whereas a pentamer of gH/gL/UL128/UL130/UL131A is selectively required for infection of epithelial, endothelial, and some myeloid-lineage cells, but not of fibroblasts. Both complexes are of considerable interest for vaccine and immunotherapeutic development but present a conundrum: gH/gL-specific antibodies have moderate potency yet neutralize CMV entry into all cell types, whereas pentamer-specific antibodies are more potent but do not block fibroblast infection. Which cell types and neutralizing activities are important for protective efficacy *in vivo* remain unclear. Here, we present evidence that certain CMV strains have evolved polymorphisms in gO to evade trimer-specific neutralizing antibodies. Using luciferase-tagged variants of strain TB40/E in which the native gO is replaced by gOs from other strains, we tested the effects of gO polymorphisms on neutralization by monoclonal antibodies (mAbs) targeting four independent epitopes in gH/gL that are common to both trimer and pentamer. Neutralization of fibroblast entry by three mAbs displayed a range of potencies that depended on the gO type, a fourth mAb failed to neutralize fibroblast entry regardless of the gO type, while neutralization of epithelial cell entry by all four mAbs was potent and independent of the gO type. Thus, specific polymorphisms in gO protect the virus from mAb neutralization in the context of fibroblast but not epithelial cell entry. No influence of gO type was observed for protection against CMV hyperimmune globulin or CMV-seropositive human sera, suggesting that antibodies targeting protected gH/gL epitopes represent a minority of the polyclonal neutralizing repertoire induced by natural infection.

## 1. Introduction

Human cytomegalovirus (CMV) is transmitted primarily through saliva and urine and infects a relatively high prevalence of human populations worldwide. CMV infections can cause pneumonitis, retinitis, gastrointestinal disfunction, encephalitis, myocarditis, and mortality in immunocompromised patients, often leading to graft rejection in solid organ transplant recipients [1]. CMV is also the most common viral infection in utero, affecting 0.05–1% of all pregnancies in the United States and Europe and 1–2% in developing countries such as India and Brazil [2,3,4]. Transplacental transmission of CMV can occur during primary maternal infection, or in the context of nonprimary infections, which include reactivation from latency or superinfection by different CMV strains. However, the rate of maternal–fetal transmission is higher for primary vs. nonprimary infections [5]. The most common disability associated with congenital infection is sensorineural hearing loss, affecting 50% of symptomatic and 10–15% of asymptomatic infants [6].

Treatment options for congenital CMV infections are limited. Currently approved antivirals are associated with dose-limiting hematologic or renal toxicities, and consequently their use is proscribed during pregnancy [7]. However, naturally acquired immunity to CMV is both protective and beneficial, and CMV hyperimmune globulin (HIG, IgG isolated from CMV-seropositive blood products) treatment shows benefits in ameliorating CMV disease in transplant patients and in preventing and treating congenital CMV infections [8,9,10]. Consequently, humoral immunity, whether vaccine-induced or passively acquired, has potential value in the development of effective therapy or prophylaxis of congenital CMV disease.

CMV infects a variety of cell types *in vivo*, and while initial virion attachment is believed to occur via interactions between cell surface glycosaminoglycans and a heterodimer of glycoproteins M and N (gM/gN), subsequent entry mechanisms are cell type-dependent and mediated by different glycoprotein complexes on the viral envelope [11,12,13]. Thus, whereas entry into fibroblasts requires glycoprotein B (gB) and a trimeric complex (trimer) of glycoproteins H, L, and O (gH/gL/gO), entry into epithelial cells, endothelial cells, and certain myeloid-lineage cells requires the trimer plus an additional pentameric complex (PC) composed of gH/gL plus UL128, UL130, and UL131A [14,15,16,17,18,19,20]. Consequently, antibodies targeting epitopes in gM/gN, gB, gH/gL, or gO have broad neutralizing abilities, while antibodies targeting epitopes specific to the PC only neutralize CMV infection of those cell types that require the PC for viral entry [21,22,23,24,25,26].

During fibroblast entry, the gH/gL/gO trimer interacts with its receptor, platelet-derived growth factor receptor-alpha (PDGFRα) [27]. The gO component, encoded by the *UL74* open reading frame, is heavily glycosylated and highly polymorphic; consequently, CMV strains cluster into eight distinct gO genotypes with the greatest sequence variability residing in the first 100 residues [28,29,30,31].

In previous studies [32], we observed that certain monoclonal antibodies (mAbs) specific to either a linear epitope in gH, or to a conformational epitope formed by gH and gL, neutralized entry of certain CMV strains into epithelial cells, thus confirming effective mAb/epitope engagement, but not into fibroblasts. As the trimer is required for entry into fibroblasts, and as the trimer contains gO, these observations led to the hypothesis that certain polymorphic versions of gO may have the ability to impair the neutralizing efficacy of mAbs targeting epitopes in gH or gH/gL. That this interference is specific for fibroblast and not epithelial cell entry follows from the fact that the PC, which lacks gO, is important for epithelial but not fibroblast cell entry.

Evidence in support of this hypothesis, at least with regard to the linear gH epitope, has been recently reported [33]. In the present work, chimeric viruses differing only in their gO sequences were constructed and used to evaluate the ability of gO proteins representing six different gO subtypes to influence the neutralizing activities of mAbs targeting: (i) four distinct gH/gL epitopes; (ii) one gB epitope; and (iii) polyclonal antibodies induced by natural infection. The results revealed that the gO type had little influence on epithelial entry neutralization by any of the antibodies tested, nor on the ability of the gB-specific mAb or of polyclonal antibodies to neutralize fibroblast entry. In contrast, one gH/gL-specific mAb failed to neutralize fibroblast entry regardless of the gO type, while fibroblast entry neutralizing potencies of three additional mAbs targeting distinct gH or gH/gL epitopes were profoundly influenced by the gO subtype. This inhibitory activity was further mapped to the highly polymorphic N-terminal domain of gO.

## 2. Materials and Methods

### 2.1. Antibodies

**Figure 1:**
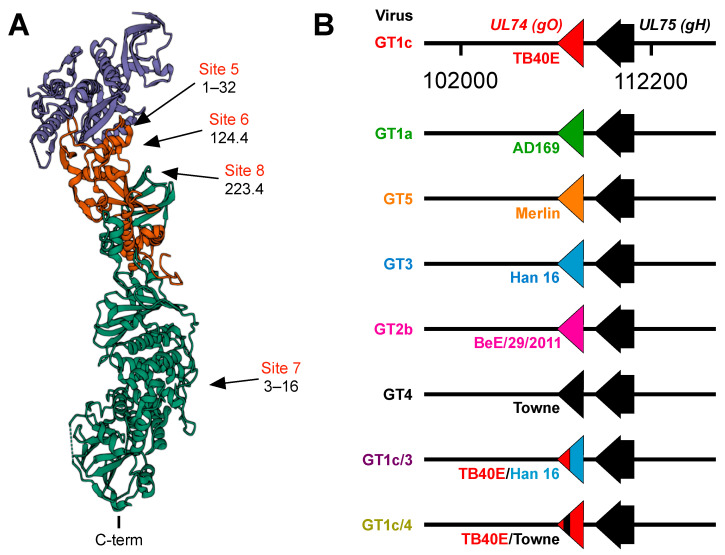
(**A**) Structure of the CMV trimer illustrating relevant immunogenic sites. The CMV trimer, as determined by cryoelectron microscopy [35], indicates the locations of gO (blue), gL (red), and gH (green) with the C-terminal transmembrane domain of gH oriented at the bottom. Approximate locations of immunogenic sites and the mAbs that target each site were inferred from mAb-gH/gL or mAb-trimer complexes characterized by negative staining electron microscopy [34] and cryoelectron microscopy [35]. (**B**) Schematic illustration of chimeric viruses. *UL74* sequences encoding TB40/E gO in the parental virus GT1c were fully or partially replaced by *UL74* sequences from the indicated CMV strains. All other sequences, including *UL75* (encoding gH), are of the background strain TB40/E.

Table 1 lists the characteristics of antibodies used in these studies, and Figure 1A shows approximate locations of their binding sites within the gH/gL/gO trimer [34,35]. Rabbit mAbs 223.4 and 124.4, specific for gH or gH/gL epitopes, were derived following immunization of a rabbit with an epitheliotropic variant of CMV strain AD169 [34]. Human mAbs 3–16 and 1–32, specific gH/gL epitopes, were isolated from memory B cells from healthy CMV-positive individuals [34]. TRL345 (Trellis Bioscience, Redwood City, CA, USA), a gift from Laurence Kauvar, is a human mAb targeting the AD-2 (site I) epitope of gB [36]. HIG (CytoGam^®^) was a gift from CSL Behring, King of Prussia, PA, USA. Sera were acquired from two normal healthy adults with CMV seropositivity confirmed by gB-ELISA [37]. Research conducted with human sera was approved by the Virginia Commonwealth University Committee for the Conduct of Human Research.

### 2.2. Cells and Viruses

Human MRC-5 lung fibroblasts (ATCC CCL-171) and ARPE-19 retinal pigment epithelium epithelial cells (ATCC CRL-2302) were obtained from ATCC and cultured in Dulbecco’s modified Eagle’s Minimum Essential Medium (Gibco/Thermo Fisher, Waltham, MA, USA) with 10% fetal bovine serum (Gemini) and 1% Penicillin Streptomycin Glutamine (Gibco/Thermo Fisher, Waltham, MA, USA).

Figure 1B illustrates the chimeric viruses used in these studies. All viruses were based on GT1c, a variant of the CMV strain TB40/E derived from the bacterial artificial chromosome (BAC) clone TB40-BAC4 [38] that had been modified to encode firefly luciferase expressed from an SV40 promoter, as described previously [39]. Previous reports [40,41] describe the construction of chimeric viruses GT4, GT1a, GT2b, GT3, and GT5 in which *UL74* sequences encoding gO in the parental strain GT1c were fully replaced by *UL74* sequences from strains Towne, AD169, BE/29/2011, or Han16, respectively, and chimera GT1c/3, which contains *UL74* sequences encoding amino acids 1–107 from strain TB40/E with the remaining 111 C-terminal amino acids from strain Han16. Genome sequences were obtained using GenBank accession numbers for CMV strains AD169 (X17403.1), Towne (GQ121041.1), BE/29/2011(KP745672.1), Han16 (JX512204.1), Merlin (NC_006273.2), and for the TB40-BAC4 variant of strain TB40/E (EF999921).

Chimera GT1c/4 was constructed using GalK recombineering [42] and encodes gO from strain TB40/E, except for residues 31–67, which are replaced by gO residues 31–64 from strain Towne. To construct GT1c/4, DNA from BAC clone GT1c was purified using a NucleoBond BAC 100 purification kit (Macherey-Nagel, Allentown, PA, USA) and transformed into competent *E. coli* strain SW102 cells using a Gene Pulser X cell electroporation system (Bio-Rad, Hercules, CA, USA). Colonies containing BAC GT1c were isolated by selection on plates containing 12.5 µg/mL chloramphenicol. Using plasmid pGalK [42] as a template, a cassette encoding GalK flanked by 50-bp homologies to target sequences in the BAC was PCR amplified using primers UL74-galK-FW (TTATATCACTGACTGTCCTGTTATTTTCTATAATAAACTGTAAGGTCGTTACGACTCACTATAGGGCGAATTGG3) and UL74-galK-RV (ATAGTAAGATTTTTAACGTGTTGCCTAGTCATATTGAAGTATTTTGTATAGCTATGACCATGATTACGCCAAGC). The product was digested with DpnI (New England Biolabs, Ipswich, MA, USA) then isolated by electrophoresis on 0.9% SeaPlaque agarose (Lonza, Basel, Switzerland). The desired 1.5-kb product was excised and extracted using the MinElute Gel Extraction kit (Qiagen, Hilden, Germany). Competent *E. coli* SW102 cells containing BAC GT1c were prepared as described [42] following incubation at 42 °C for five minutes to induce recombinase expression. Cells were transformed with 100 ng of gel-purified PCR product by electroporation at 25 mF and 1.75 kV and GalK-positive colonies were isolated on plates containing 0.2% D-galactose (Thermo Fisher, Waltham, MA, USA) and 12.5 µg/mL chloramphenicol. Correct *galK* insertion was confirmed by PCR and targeted sequencing (eurofins).

Using Towne DNA as a template, a PCR product containing *UL74* sequences from strain Towne flanked by 50-bp homologies to sequences flanking the *galk* insertion was amplified using primers UL74-Towne-FW (TCTAAATTATTCTTTATTATATCACTGACTGTCCTGTTATTTTCTATAATAAACTGTAAGATCGCGGTAGCGCGTTTTCGAGTAAAGAGTCAGAAAGCAAAAGAGGAAGAGAGGCAACTA) and UL74-Towne-RV (GGTCATATTCATAGTAAGATTTTTAACGTGTTGCCTAGTCATATTGAAGTATTTTGTATAATCACCTGTTTTTGACGCTAGTTCTTGCAGTATACGTAATTTTAGTTGCCTCTCTTCCTC). The product was purified using the MinElute PCR purification kit (Qiagen, Hilden, Germany) and transformed as above into induced/competent *E. coli* SW102 cells containing the *galK* insertion mutant of BAC GT1c. Clones in which *galK* was replaced by Towne *UL74* sequences were isolated by counter-selection on plates containing 0.2% 2-Deoxy-D-galactose (Alfa Aesar, Haverhill, MA, USA) and 12.5 µg/mL chloramphenicol. Correct insertion of Towne sequences in the resulting BAC clone, designated GT1c/4, was confirmed by PCR and targeted sequencing (eurofins). GT1c/4 BAC DNA was prepared as described above and transfected into MRC-5 cells using effectene transfection reagent (Qiagen, Hilden, Germany), as previously described [41,43]. Virus stocks were derived from infected cell culture supernatants, adjusted to 0.2 M sucrose, and stored in liquid nitrogen. Viral titers were determined using MRC-5 cells as described [43].

### 2.3. Immunofluorescence-Based Neutralization Assays

Approximately 300 PFU of each chimeric virus were incubated with mAbs at a final concentration of 100 μg/mL for one hour (h) at 37 °C, then transferred to black-walled clear-bottom 96-well plates (Corning) containing confluent MRC-5 or ARPE-19 cell monolayers. Immunofluorescence staining was performed as previously described [44]. After incubation for 48 h at 37 °C the cells were fixed with 3% formaldehyde in phosphate-buffered saline (PBS, Fisher Bioreagents) at room temperature (RT) for 30 min (min), permeabilized with 0.5% Triton-×100 in PBS on ice for 20 min, and blocked with 20% fetal bovine serum in 1× PBS for 30 min. Cells were then incubated for 1 h with mouse monoclonal antibody MAB810 (1:600; EMD MilliporeSigma, Burlington, MA, USA) specific for an epitope common to the CMV Immediate Early 1 and Immediate Early 2 (IE1/2) proteins, washed with blocking buffer, and incubated with Alexa Fluor 488-conjugated goat anti-mouse IgG (1:200; Life Technologies/Thermo Fisher, Waltham, MA, USA) for one hour at RT. Images of fluorescently labeled cells were obtained using a Nikon Eclipse TS100 microscope equipped with a digital camera and NIS-Elements version 4.0.

### 2.4. Luciferase-Based Neutralization Assays

Serial dilutions of each virus stock were used to infect black-walled clear-bottom 96-well plates containing confluent MRC-5 or ARPE-19 cell monolayers. After incubation for 48 h at 37 °C, 100 μL of Steady-Glo Luciferase substrate (Promega, Madison, WI, USA) was added to each well and after incubation at RT for an additional 10 min, relative luminescence units (RLU) were measured using a Synergy^TM^ HTX Multi-Mode Microplate Reader (BioTek, Winooski, VT, USA). The amounts of each virus stock needed to generate a signal of 10,000–20,000 RLU at 48 h post-infection (hpi) were determined from the resulting dose–response curves. This amount of each virus was then mixed with serial dilutions of mAbs, HIG, or human sera, incubated for 1 h at 37 °C, and transferred in triplicate to 96-well cultures containing confluent MRC-5 or ARPE-19 monolayers. After 48 h RLU were measured as above.

### 2.5. Statistical Analyses

The 50% inhibitory concentration (IC_50_) values were determined using Prism 5 (GraphPad Software, Inc., San Diego, CA, USA) as the inflection points of four-parameter curves fitted by non-linear regression to plots of mean relative fluorescent units (from triplicate wells) vs. Log (antibody concentration) as described previously [45]. Where neutralization did not reach 100%, IC_50_ values were reported as the antibody concentration at which the fitted curve intersected the 50% inhibition line. Where neutralization did not reach 50%, IC_50_ values were reported as >50 μg/mL, the highest antibody concentration used. Luciferase-based assays were repeated at least twice with similar results.

## 3. Results

In a previous study [34] a panel of mAbs was used to define antigenic sites in the PC containing neutralizing epitopes. Antibodies to sites 1–4 were determined to be specific to the PC because they bound to recombinant PC but not to gH/gL and neutralized epithelial but not fibroblast cell entry, while antibodies to sites 5–8 were determined to be gH/gL-specific because they bound to both the PC and gH/gL. Based on negative staining electron microscopy, sites 5–8 mapped either to membrane proximal or distal regions of the trimer (Figure 1A). Antibodies targeting sites 5–7 neutralized epithelial cell entry by a wide range of CMV strains, while those to site 8 exhibited considerable strain specificity [34]. Despite neutralizing epithelial cell entry, mAbs to sites 5 and 8 failed to neutralize fibroblast entry of an epitheliotropic variant of strain AD169 [34]. In subsequent studies site 6 mAbs were found to neutralize epithelial but not fibroblast cell entry of an epitheliotropic variant of strain Towne [32]. These findings raised the possibility that certain gH/gL epitopes may be accessible to mAb binding in the context of the PC, resulting in neutralization of epithelial cell entry, while in the context of the trimer access might be prevented or precluded by certain forms of gO; thus, selectively disrupting their ability to neutralize fibroblast entry. To address this hypothesis we utilized chimeric viruses in which *UL74* sequences (encoding gO) from various CMV strains were genetically swapped for the native *UL74* sequences in parental virus GT1c, a BAC-cloned virus derived from CMV strain TB40/E [40] (Figure 1B).

### 3.1. Towne gO Inhibits the Ability of mAbs Targeting Epitopes in Sites 6 and 8 of gH/gL to Neutralize Fibroblast Entry

**Figure 2:**
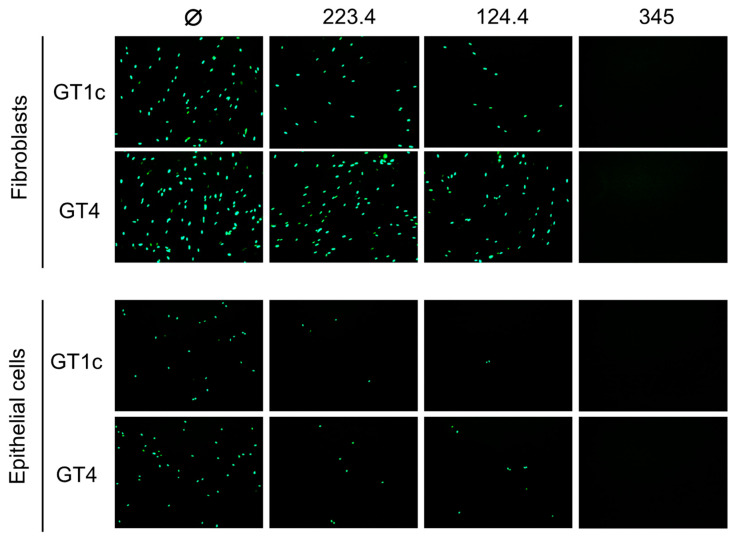
Strain Towne gO impairs the fibroblast entry neutralizing activities of mAbs targeting epitopes in gH/gL. Viruses GT1c (encoding TB40/E gO) and GT4 (encoding Towne gO) were incubated for 1 h at 37 °C in medium (Ø) or medium containing 100 µg/mL of mAbs 223.4, 124.4, or TRL345, then added to MRC-5 fibroblast or ARPE-19 epithelial cell monolayers in 96-well plates. Following incubation at 37 °C for 48 h, infected cells were detected by immunofluorescent staining for the CMV IE1/2 proteins. Representative images are shown.

In an initial pilot study to validate our hypothesis, mAb neutralizing activities were evaluated against the parental GT1c virus, containing the *UL74* sequence from TB40/E, or against GT4, a variant derived from GT1c in which the native *UL74* was replaced with *UL74* from strain Towne (Figure 1B) [40]. The two viruses were incubated with mAb 223.4, which targets a linear gH epitope in site 8, with mAb 124.4, which targets the gH/gL conformational epitope in site 6, or with mAb TRL345, which targets a highly conserved linear epitope in gB (Table 1). Following incubation to allow virion/mAb binding, replicate mixtures were added to monolayers of MRC-5 fibroblasts or ARPE-19 epithelial cells. Viral entry was assessed at 48 hpi by immunofluorescence staining for the CMV IE1/2 proteins. Both 124.4 and 223.4 mAbs significantly reduced fibroblast entry of virus GT1c, yet neither strongly impacted fibroblast entry of virus GT4. In contrast, epithelial entry of both viruses was similarly reduced by mAbs 124.4 and 223.4, and both viruses were fully neutralized by the gB-specific mAb TRL345 regardless of the target cell type (Figure 2). As GT4 and GT1c do not differ with respect to the epitopes targeted by these antibodies but differ only with respect to the gO type, these results suggest that Towne gO is able to impair the neutralizing activities of mAbs 124.4 and 223.4 in the context of fibroblast but not of epithelial cell entry.

### 3.2. Quantitative Luciferase-Based Assays Confirm That Towne gO Inhibits the Ability of mAbs Targeting gH/gL Epitopes in Sites 6, 7, and 8 to Neutralize Fibroblast Entry

**Figure 3:**
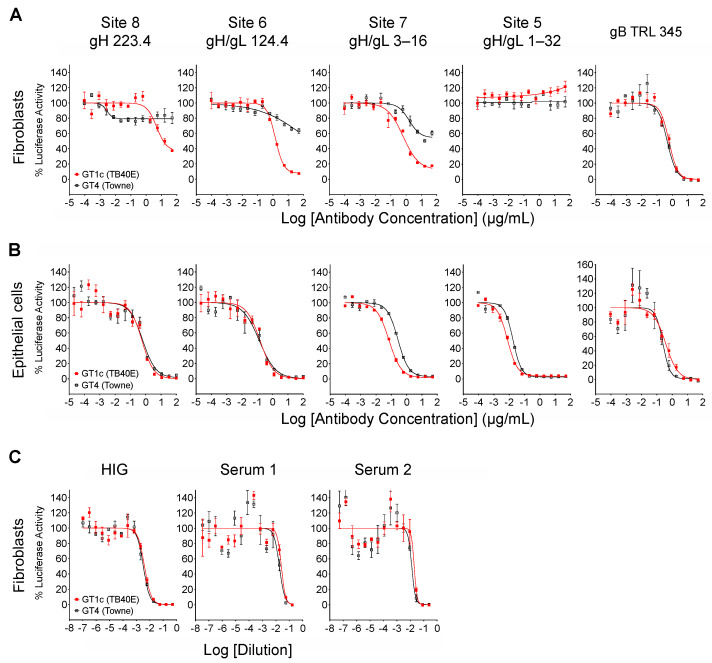
Towne gO impairs fibroblast entry neutralization by mAbs targeting three independent gH/gL epitopes but does not impact net neutralizing activities of anti-CMV antibodies induced by natural infection. Viruses GT1c or GT4 were incubated for 1 h at 37 °C with serial dilutions of mAbs, HIG, or human sera, then added in triplicate to 96-well cultures of MRC-5 fibroblasts (**A**,**C**) or ARPE-19 epithelial cells (**B**). After incubation at 37 °C for 48 h, luciferase activity in each well was measured and the % of maximal luciferase activity (means of triplicate wells) was plotted vs. log of antibody concentration or dilution.

When measured at 48 hpi the luminescence signal produced by the luciferase reporter cassette encoded by viruses GT4 and GT1c [40] is proportional to viral entry [46]. Luciferase-based neutralizing assays were therefore performed to quantitatively evaluate the impact of Towne gO on the neutralizing activities of mAbs. In addition to mAbs 124.4 and 223.4 targeting sites 6 and 8 (described above), mAb 1–32 targeting site 5 and mAb 3–16 targeting site 7 were evaluated. Results clearly show that Towne gO profoundly impaired the ability of mAbs 223.4, 124.4, and 3–16 to block viral entry into fibroblasts (Figure 3A, Table 2). At the highest concentration tested (100 µg/mL) 223.4 reduced GT1c infectivity by 63% but only reduced GT4 by 21%; similarly, 124.4 reduced GT1c infectivity by 92% and GT4 by 36%, while 3–16 reduced GT1c infectivity 88% and GT4 by 46% (Table 3). Consistent with our initial finding that mAb 1–32 neutralizes epithelial but not fibroblast entry of a variant of strain AD169 [34], 1–32 failed to reduce fibroblast infectivity of either GT1c or GT4 even at 100 µg/mL (Figure 3A, Table 2). In contrast, the gB-specific TRL345 mAb fully neutralized fibroblast infectivity of both viruses (Figure 3A, Table 2). Parallel experiments using ARPE-19 epithelial cells as targets confirmed the ability of 1–32 to neutralize epithelial cell entry but revealed no significant impact of the gO type on neutralization by 1–32 or any of the other mAbs tested (Figure 3B, Table 2).

### 3.3. Inhibition by Towne gO Is Not Sufficient to Influence the Net Neutralizing Activities of Polyclonal Antibodies Induced by Natural Infection

The neutralizing activity of HIG or sera from CMV-infected individuals is the aggregate result from antibodies targeting a broad range of neutralizing epitopes in several viral envelope glycoprotein complexes. However, antibodies to gB dominate the neutralizing activity affecting fibroblast entry [47,48,49] while antibodies to the PC dominate the activity affecting epithelial cell entry [27,50]. Although the inhibitory effects of gO are likely to be restricted to a limited number of epitopes within gH/gL, the type of gO could have an influence if a significant portion of the neutralizing activity induced by natural infection is attributable to antibodies targeting these gH/gL epitopes. Thus, to determine if gO type alters the neutralizing potency of polyclonal antibody mixtures induced by natural CMV infection, GT1c and GT4 were again used in fibroblast-based assays to evaluate the neutralizing activities of HIG, as well as sera from two individual CMV-seropositive donors. For all three samples, the neutralization curves generated with GT1c and GT4 were superimposable (Figure 3C), indicating that any influence of Towne gO on inhibiting the activity of antibodies specific to gH/gL epitopes is not sufficient to perturb the net neutralizing effects of these complex polyclonal antibody mixtures.

### 3.4. Inhibition Extends to Other gO Types and Is Epitope-Specific

**Figure 4:**
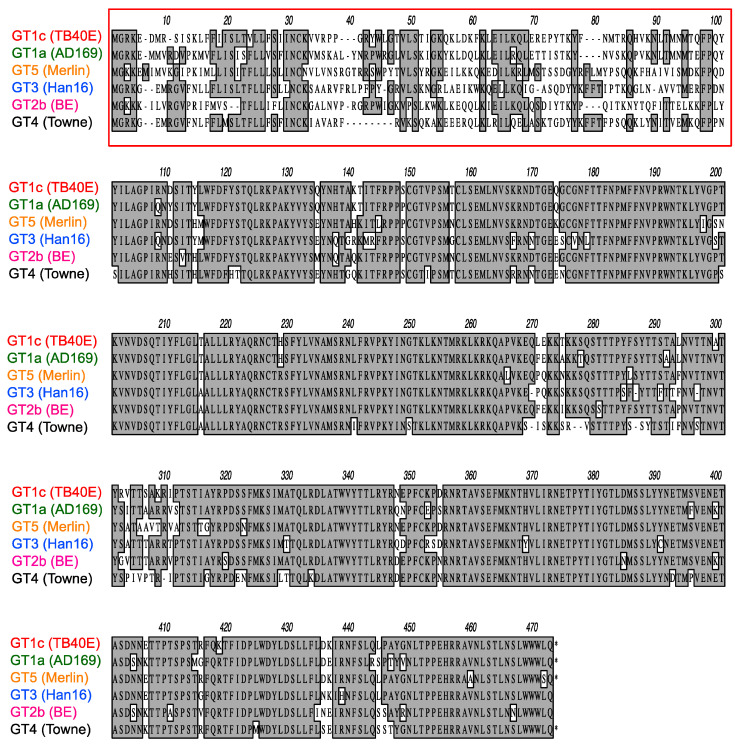
Polymorphisms of gO amino acid sequences encoded by diverse CMV strains. Predicted gO amino acid sequences from CMV strains TB40/E, AD169, Merlin, Han16, BE/29/2011, and Towne were aligned using clustalW. Numbers refer to amino acid positions in Merlin gO; the red box indicates the most highly polymorphic region.

As illustrated in Figure 4, CMV gO exhibits significant strain polymorphism, particularly in the N-terminal 100 residues. In addition to strain Towne, prior studies indicated that fibroblast entry of strain AD169 is resistant to mAbs targeting gH/gL epitopes in sites 5 and 8 [32,34]. To assess the extent to which gO types from AD169 or other CMV strains may influence neutralizing activities, additional chimeric viruses were constructed in which *UL74* in GT1c was replaced with *UL74* sequences from CMV strains AD169 (GT1a), Merlin (GT5), Han 16 (GT3), or BE/29/2011 (GT2b) (Figure 1B). Luciferase-based neutralization assays were performed with the additional chimeric viruses. Among the panel of chimeric viruses, GT1c, encoding TB40/E gO, was consistently the most sensitive to mAbs.

**Figure 5:**
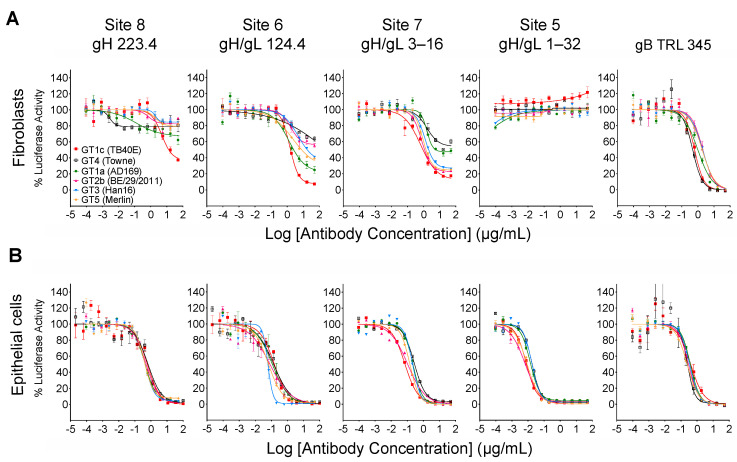
Other gO types influence fibroblast entry neutralization by gH/gL-specific mAbs in an epitope-specific manner. Four additional TB40/E-background chimeric viruses encoding gOs from strains AD169, Merlin, Han16, or BE/29/2011 were used in fibroblast (**A**) or epithelial cell (**B**) entry neutralizing assays as described in Figure 3.

223.4, 124.4, and 3–16, while GT4 (encoding Towne gO) was consistently among the most resistant to all three antibodies (Figure 5A). Interestingly, some gO types exhibited differential/epitope-specific resistance. For example, viruses GT5, GT3, and GT2b (encoding gOs from Merlin, Han 16, and BE/29/2011, respectively) were highly resistant to 223.4, moderately resistant to 124.4, and relatively sensitive to 3–16, while virus GT1a (encoding gO from AD169) was sensitive to 124.4 but relatively resistant to 223.4 and 3–16 (Figure 5A). In contrast, mAb 1–32 was ineffective in neutralizing fibroblast entry regardless of gO type, even at the highest concentration of 100 µg/mL (Figure 5A). As before, the gB-specific TRL345 mAb fully neutralized fibroblast infectivity of all six chimeric viruses (Figure 5A), and parallel experiments using ARPE-19 epithelial cells as targets revealed no significant impact of the gO type on neutralization by the five mAbs tested (Figure 5B, Table 2).

### 3.5. Inhibition by Towne gO Maps to the Polymorphic N-Terminal Region of gO

**Figure 6:**
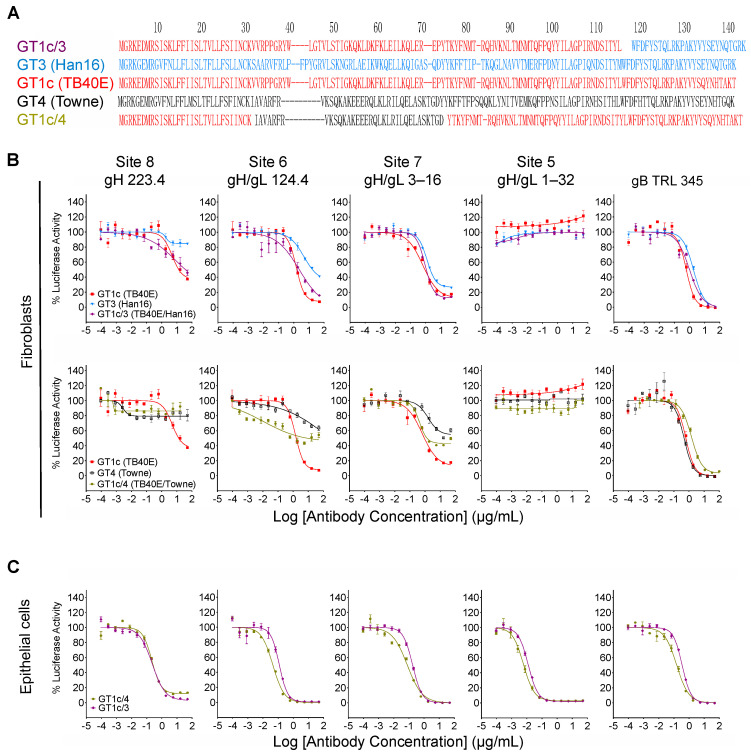
Inhibitory activity maps to the polymorphic N-terminal region of gO. (**A**) Predicted gO amino acid sequences from CMV strains TB40/E (red), Towne (black), and Han16 (blue) were aligned using clustalW to illustrate the respective sequences in intrastrain gO chimeras GT1c/3 and GT1c/4. Intrastrain gO chimeric viruses GT1c/3 and GT1c/4 were used in fibroblast (**B**) or epithelial cell (**C**) entry neutralizing assays as described in Figure 3. Data from chimeras encoding full-length gOs that comprise parts of each intrastrain gO chimera are included for comparison.

While the overall amino acid identity between TB40/E and Towne gO is 76%, the majority of polymorphisms lie within the first 100 residues, where the amino acid identity is only 43% (Figure 4, red box). To determine if this region is important for inhibitory activity, two gO partial chimeric viruses, GT1c/3 and GT1c/4 (Figure 1B), were evaluated. Virus GT1c/3 encodes the N-terminal 107 residues from TB40/E gO (Figure 6A, red) with the remainder of the gO protein from Han 16 (Figure 6A, blue), and virus GT1c/4 encodes N- and C-terminal gO sequences from TB40/E (Figure 6A, red) but residues 31–67 from the homologous region (residues 31–64) of Towne (Figure 6A, black). Luciferase-based neutralization assays were performed on the two partial chimeras and compared to viruses expressing complete gO sequences from strains Towne, Han 16, or TB40/E. Sensitivity or resistance of the partial chimeras to mAb neutralization of fibroblast entry was determined by their N-terminal gO sequences. Thus, virus GT1c/3, encoding the N-terminal gO sequence from TB40/E, remained sensitive to mAbs 223. 4 or 124.4 to an extent similar to that of virus GT1c, encoding the full-length native TB40/E gO. In contrast, virus GT1c/4, in which N-terminal sequences of gO were replaced by residues from strain Towne, was resistant to mAbs 223.4, 124.4, and 3–16 (Figure 6B). As in previous experiments, mAb 1–32 could not neutralize fibroblast entry of GT1c/3 or GT1c/4 and no significant impact of the gO type was observed on neutralization by the gB-specific mAb TRL345 or on neutralization of epithelial cell entry by the five mAbs tested (Figure 6B,C).

## 4. Discussion

CMV is among the most common pathogens that cause opportunistic diseases in immunocompromised individuals such as transplant and AIDS patients [51]. Disease is commonly associated with reactivation of latent CMV, re-infection with different CMV strains (superinfection), or primary infection arising from transplantation of infected tissues or organs [52,53]. In the congenital infection setting over two-thirds of newborns infected in utero are born to women who were infected prior to pregnancy [54,55,56], indicating that preexisting cellular and humoral maternal immunity does not fully prevent placental transmission [57,58,59,60,61]. A study in rhesus CMV demonstrated that superinfection requires evasion of CD8+ T cell recognition of infected cells by viral inhibitors of major histocompatibility complex class I expression [62]. While the importance of CMV evasion of antibody mechanisms is less understood, polymorphisms in viral glycoproteins, particularly those directly or indirectly impacting neutralizing epitopes, suggest that evolutionary pressures have favored mutations to evade recognition by antibodies targeting specific epitopes.

In the current study, gO sequences from various CMV strains were found to act in *trans* to protect neutralizing epitopes in the gH/gL dimer. These results are consistent with earlier observations that resistance to inhibition of spread by gH-specific mAbs C2 and 14–4b correlates with strain differences in gO but not gH [31], and that deletion of the *UL74* gene enhances sensitivity to inhibition of spread by gH-specific mAb 14–4b and gB-specific mAb C23 [63]. In a more recent study, Day et al. found that a chimeric virus expressing gO from strain TB40/E in the strain TR background was more sensitive to fibroblast entry neutralization by gH-specific mAbs 14–4b and AP86-SA4 than viruses expressing the native gO of strain TR or a heterologous gO from strain Towne [33]. As AP86-SA4 recognizes a linear epitope in site 8 [33], the results for AP86-SA4 are therefore analogous to and consistent with our results for mAb 223.4. Although 14–4b targets a membrane-proximal epitope [64] it is not known if this corresponds to site 7 or comprises an epitope that is not among those defined by the mAbs shown in Figure 1A. In any case, the present study extends the phenomenon of gO-mediated epitope protection in *trans* to four distinct gH/gL epitopes and gOs representing six different subtypes. Moreover, the results from gO chimeras determined that the region of gO that confers epitope protection in *trans* corresponds to the highly polymorphic N-terminal 98 amino acids, with residues 31–64 from gO of strain Towne being sufficient to confer resistance.

Based on negative staining electron microscopy of mAb-gH/gL complexes [34], site 5 is near the apical/membrane-distal end of gH/gL (Figure 1A). A recent cryoelectron microscopy structure of mAb 1–32 bound to site 5 in the pentamer places site 5 at the gL/UL128 interface [65]. As gO covers a larger footprint on gL than UL128 [35], the 1–32 epitope may simply be occluded by gO in the context of the trimer. However, Kschonsak et al. also noted that in the pentamer gL residues A131 to V151 form a helix at the interface and disulfide bond with UL128, while in the trimer the same residues interface with gO but the helix is disrupted to form a loop [35]. Thus, it is possible that gO perturbs the local gL structure sufficiently to alter the topology of the nearby 1–32 epitope. In either case, that 1–32 fails to neutralize fibroblast entry by all gO chimera viruses tested indicates that this is a universal effect of gO and is not specific to certain gO types.

Negative staining electron microscopy and mAb binding competition experiments indicate that sites 5 and 6 are adjacent or partially overlap [34]. Site 8 presumably lies slightly more distal from gO than sites 5 and 6 (Figure 1A), although the exact location of the linear epitope recognized by mAb 223.4 is uncertain because it lies within an unstructured N-terminal region of gH [35]. Despite the apparent close proximity of sites 6 and 8 to site 5, fibroblast entry neutralizing activities of mAbs targeting sites 6 and 8 were differentially affected by different gO types. This may again suggest occlusion of sites 6 or 8 by certain gOs due to larger footprints of their gO-gL interfaces, or perhaps differential perturbation gL structure by different gOs. Site 7, however, appears to be far more membrane-proximal based on negative staining electron microscopy [34], and yet remains differentially influenced by gO polymorphisms. This large physical distance from gO suggests that occlusion of site 7 by some gO types is unlikely. Thus, conformational changes in gH/gL induced by binding of certain gO types may act to disrupt mAb binding to distal sites in the trimer, or alternatively, may permit mAb binding but mitigate their neutralizing effects, for instance by promoting downstream interactions between trimer and gB despite the presence of bound mAbs. Trimer-mAb binding studies as well as high resolution structures of mAb-binding epitopes in trimers containing different gO types are needed to resolve these mechanistic details.

While it is possible that the gO type could alter the levels of trimer in virions, thus impacting the neutralizing activities of trimer-specific neutralizing antibodies, previous studies have shown that gO-chimeric viruses retain the ability to infect epithelial cells and fibroblasts [41] and that gH and gO levels in virions of viruses GT3, GT4, and GT5 are comparable to those of the parental virus GT1c [40,41]. Similarly, Day et al. showed that replacing gO in strain TR or Merlin backgrounds does not alter trimer/pentamer ratios compared to the parental viruses [33]. Thus, the impact of gO polymorphisms on mAb neutralizing activities does not appear to be related to variations in virion trimer levels.

The N-terminus of gO is important for trimer binding to its cellular receptor PDGFRα on fibroblasts [28,66,67]. Although polymorphisms in gO may affect some of the recently identified trimer-PDGFRα interaction surfaces [35], data from Brait et al. showed that PDGFRα-Fc completely inhibits fibroblast infectivity of all gO-chimeric mutants in the GT series, indicating that replacing gO from different CMV strains does not significantly alter PDGFRα binding [41]. Based on the structure of the trimer, mAbs to site 7 and perhaps also sites 6 and 8 appear unlikely to interfere with PDGFRα binding. Thus, antibodies to these sites might neutralize by disrupting trimer interactions with gB or by interfering with conformational changes in gH/gL, driven by PDGFRα binding, that serve to promote trimer-gB interactions leading to membrane fusion. Further studies, particularly to assess the impact of gOs from different strains on mAb-trimer binding, will help to elucidate these mechanisms.

Like gO, gN is heavily glycosylated, exhibits extensive amino acid sequence polymorphism, and mediates neutralizing epitope protection in *trans*. The mechanism of protection has been linked to glycosylation, as mutations in gN that reduce its glycosylation enhanced sensitivity to antibodies targeting epitopes in gB and gH, in addition to gN [68]. Recent cryoelectron microscopy studies on the prefusion structure of gB revealed a potential interaction between prefusion gB and the membrane proximal side of gH [69], suggesting that the influence of certain gO types could potentially extend to neutralizing epitopes in gB. This was not the case, however, at least for one linear epitope in the AD-2 site of gB, as mAb TRL345, specific to this epitope, neutralized both fibroblast and epithelial infectivity of all six chimeric gO viruses with similar potencies. The possibility remains, however, that gO polymorphisms influence neutralizing epitopes in gB more proximal to a proposed gB-trimer interface [70,71]. However, given that fibroblast entry neutralizing activity of HIG or CMV-positive human sera was also not influenced by gO subtype, and that much of the neutralizing activity in these polyclonal mixtures is mediated by gB- or gM/gN-specific antibodies [47,72,73], it is probable that gO-mediated protection applies primarily to gH/gL epitopes; consequently, antibodies to gB, gM, or gN epitopes that are not influenced by gO may dominate, while the contributions of gO-protected gH/gL epitopes comprise a minority of the aggregate neutralizing activity of these polyclonal antibody mixtures. Whether gO-mediated resistance in *trans* involves glycan shielding, as proposed for gN [68], or extends to neutralizing epitopes in other CMV glycoproteins, such as gB, gM, or gN, will require further study.

Protection in *trans* by gO may allow escape from neutralizing antibodies targeting gH/gL epitopes induced by a previous infection, and therefore provide advantages during reinfection. As the epitopes are presumably protected regardless of their polymorphisms, this mechanism of antibody evasion would be equally effective in the context of prior immunity induced by strains with the same or similar epitopes or by the same strain, as occurs when CMV reactivates from latency. Why certain strains such as Towne have acquired this mechanism of neutralizing antibody evasion while others, such as TB40/E have not, remains a mystery. However, it is possible that the TB40/E gO provides other advantages; for example, it may protect other epitopes in gH/gL that have not as yet been identified.

While the importance or clinical relevance of protecting epitopes in gH/gL is not known, that the linear epitope at site 8 is polymorphic [32] suggests that protecting this epitope from antibody recognition conferred an advantage at some point in CMV’s evolution. However, given that the neutralizing potency of polyclonal antibodies induced by CMV infection is not measurably affected by gO type, such advantages may be subtle or limited to certain circumstances *in vivo*. Consequently, their impact may only manifest in the context of population dynamics over multiple host infections. Nevertheless, that CMV has evolved elaborate mechanisms to avoid neutralization by mAbs targeting gH/gL, and specifically to protect fibroblast and not epithelial cell entry, suggests that fibroblast infection may have special importance for the natural history of CMV infections.

## Figures and Tables

**Table 1:** Antibody properties.

*Type*	*Antibody*	*Target*	*Species*	*Epitope* ^1^	*Location* ^4^
monoclonal	124.4 ^2^	gH/gL site 6	rabbit	C	membrane distal
	223.4 ^2^	gH site 8	rabbit	L	membrane distal
	3–16 ^2^	gH/gL site 7	human	C	membrane proximal
	1–32 ^2^	gH/gL site 5	human	C	membrane distal
	TRL345 ^3^	gB	human	L	
polyclonal	HIG	pan CMV	human	P	
	human serum 1	pan CMV	human	P	
	human serum 2	pan CMV	human	P	

^1^ nature of epitopes: linear (L), conformational (C), mixed/polyclonal (P); ^2^ [34]; ^3^ [36]; ^4^ epitope location relative to membrane surface, determined by negative staining electron microscopy [34].

**Table 2:** IC_50_ concentrations (μg/mL) ^1^ for mAb neutralization of fibroblast (F) or epithelial (E) cell entry.

*Virus*	*gO*	*223.4*		*124.4*		*3–16*		*1–32*		*TRL345*	
		*F*	*E*	*F*	*E*	*F*	*E*	*F*	*E*	*F*	*E*
GT1c	TB40/E	13.97	0.60	1.66	0.15	0.92	0.07	>50	0.008	0.62	0.40
GT1a	AD169	>50	0.41	2.60	0.11	5.55	0.22	>50	0.01	0.99	0.38
GT2b	BE/29/2011	>50	0.49	>50	0.08	1.15	0.11	>50	0.007	2.02	0.29
GT3	Han16	>50	0.40	17.75	0.06	2.09	0.21	>50	0.02	1.95	0.34
GT5	Merlin	>50	0.38	7.99	0.06	1.42	0.15	>50	0.009	1.86	0.24
GT4	Towne	>50	0.64	>50	0.14	>50	0.27	>50	0.02	0.47	0.25

^1^ concentration of mAb required to reduce luciferase activity by 50%; >50 indicates mAb failed to achieve 50% reduction at the highest concentration tested (50 μg/mL).

**Table 3:** Reduction in fibroblast infectivity following mAb neutralization ^1^.

*Virus*	*gO*	*Reduction in Fibroblast Infectivity (%)*		
		*223.4*	*124.4*	*3–16*
GT1c	TB40/E	S ^2^ (63)	S (92)	S (88)
GT1a	AD169	R ^3^ (33)	S (78)	R (52)
GT2b	BE/29/2011	R (17)	R (44)	S (77)
GT3	Han16	R (15)	S (65)	S (73)
GT5	Merlin	R (21)	S (72)	S (75)
GT4	Towne	R (21)	R (35)	R (46)
GT1c/3	TB40/E/Han16	R (54)	S (84)	S (88)
GT1c/4	TB40/E/Towne	R (14)	R (55)	R (57)

^1^ 100 μg/mL mAb; ^2^ sensitive (>60% reduction); ^3^ resistant (<60% reduction).

## Data Availability

Not applicable.
